# Grape‐Seed Proanthocyanidin Extract (GSPE) Modulates Diurnal Rhythms of Hepatic Metabolic Genes and Metabolites, and Reduces Lipid Deposition in Cafeteria‐Fed Rats in a Time‐of‐Day‐Dependent Manner

**DOI:** 10.1002/mnfr.202400554

**Published:** 2024-11-11

**Authors:** Romina M. Rodríguez, Leonardo Vinícius Monteiro de Assis, Enrique Calvo, Marina Colom‐Pellicer, Sergio Quesada‐Vázquez, Álvaro Cruz‐Carrión, Xavier Escoté, Henrik Oster, Gerard Aragonès, Miquel Mulero

**Affiliations:** ^1^ Nutrigenomics Research Group Department of Biochemistry and Biotechnology Campus Sescelades Universitat Rovira i Virgili (URV) Tarragona 43007 Spain; ^2^ Institute of Neurobiology Center of Brain Behavior and Metabolism University of Lübeck Marie Curie Street 23562 Lübeck Germany; ^3^ Center of Environmental Food and Toxicological Technology‐TecnATox Rovira i Virgili University Reus 43201 Spain; ^4^ Eurecat, Technology Centre of Catalunya Nutrition and Health Unit Reus 43204 Spain; ^5^ United States Department of Agriculture and The Agricultural Research Service ^(^USDA‐ARS) Arkansas Children's Nutrition Center and Department of Pediatrics University of Arkansas for Medical Science Little Rock AR 72202 USA

**Keywords:** chronobiology, circadian rhythms, liver metabolism, NAFLD/MASLD, obesity, proanthocyanidins

## Abstract

**Scope:**

Metabolic dysfunction‐associated steatotic liver disease (MASLD) is a global health issue with increasing prevalence. Polyphenols, such as grape seed proanthocyanidin extract (GSPE), are bioactive compounds present in plants and represent an interesting therapeutical approach for MASLD.

**Methods and results:**

This study questioned whether the timing of GSPE administration impacts liver diurnal metabolism and steatosis in a rat obesity model. Results from hepatic lipid profiling and diurnal metabolic gene expression and metabolomics reveal that rats fed with a cafeteria (CAF) diet show impaired glucose homeostasis and enhanced lipogenesis in the liver, contributing to liver steatosis. Chronic consumption of GSPE in the inactive or active phase is associated with beneficial effects as the restoration of rhythms of transcripts and metabolites is observed. However, only when given in the active phase, GSPE treatment decreases hepatic triglyceride levels. Using an in vitro hepatocyte model, the study identifies that catechin, one of the main phenolic compounds found in the GSPE extract, is a potential mediator in ameliorating the effects of CAF‐induced liver steatosis.

**Conclusion:**

Taken altogether, the findings show that the beneficial effects of GSPE on MASLD development depend on the treatment time.

## Graphical abstract

Visual summary of the main results of the research.

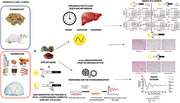

## 1 Introduction

Metabolic dysfunction‐associated steatotic liver disease (MASLD) is a disease spectrum varying from simple steatosis to steatohepatitis and cirrhosis in which insulin resistance is a pivotal pathogenetic hallmark.^[^ ^1^, ^2^ ^]^ Excess food intake and sedentary lifestyles are also associated with the growing worldwide epidemic of metabolic disorders and MASLD.^[^ ^3^, ^4^ ^]^ In addition, circadian disruption by artificial light at night and shiftwork, together with sleep deprivation, are essential features of the industrialized world that further contribute to the pathogenesis of MASLD.^[^ ^5^ ^]^

The master circadian pacemaker, the suprachiasmatic nucleus (SCN), located in the hypothalamus, is influenced primarily by light and exerts temporal control over clocks in peripheral tissues.^[^ ^6^, ^7^ ^]^ Metabolic organs such as the liver are also affected by feeding time and type of diet.^[^ ^6^, ^8^ ^]^ In mice, the intake of a high‐fat diet generates a profound reorganization of the liver clock with altered diurnal rhythms of transcripts and metabolites.^[^ ^9^ ^]^ Interestingly, induction of hepatic steatosis leads to a phase advance of core clock genes, which is further shifted to approximately 4 h in metabolic dysfunction‐associated steatohepatitis (MASH).^[^ ^9^, ^10^ ^]^

Polyphenols, particularly proanthocyanidins, are naturally occurring secondary plant metabolites found in many fruits and vegetables.^[^ ^11^, ^12^ ^]^ Proanthocyanidins possess antioxidant, antimicrobial, anti‐inflammatory, anti‐allergic, anti‐obesogenic, and vasodilatory properties.^[^ ^13^, ^14^ ^]^ Epidemiological evidence suggests that proanthocyanidin consumption reduces the risk of chronic diseases, including MASLD.^[^ ^15^ ^]^ Specifically, it has been shown that grape seed proanthocyanidin extract (GSPE), which is mainly composed of flavan‐3‐ol monomeric, dimeric, and trimeric procyanidins and shows high bioactivity,^[^ ^16^, ^17^ ^]^ for example, exhibiting hepatoprotective effects in animal models of diet‐induced obesity.^[^ ^18^, ^19^ ^]^ Evidence suggests that polyphenols may also affect circadian rhythms by influencing central and peripheral clocks mostly via transcriptional regulation.^[^ ^20^, ^21^, ^22^, ^23^, ^24^ ^]^

In this study, we questioned whether the timing of GSPE administration affects its therapeutic potential in MASLD. We addressed this question by subjecting mice to an obesogenic cafeteria (CAF) diet followed by treatment with GSPE during the beginning of the inactive (light, ZT 0) or active phase (dark, ZT12). We identified that GSPE treatment during the active phase led to higher lipid catabolism, which was followed by alterations at the transcriptional level of several glucose and lipid metabolism‐associated genes. In a circadian reporter hepatocyte model, we identified catechin, a flavanol highly abundant in GSPE, as a promising mediator of GSPE effects on the liver clock and metabolic gene expression rhythms.

## 2 Experimental Section

### 2.1 Grape Seed Proanthocyanidin Extract

GSPE was obtained from Les Dérives Résiniques et Terpéniques (Dax, France). GSPE was directly analyzed by LC‐MS/MS (Agilent Technologies, Palo Alto, CA, USA). A ZORBAX SE‐aq column (150 mm × 2.1 mm i.d., 3.5 µm particle size, Agilent Technologies) was used. The mobile phase consisted of A) 0.2 % acetic acid in water and B) acetonitrile. The gradient mode was as follows: initial conditions, 5% B; 0–10 min, 5–55% B; 10–12 min, 55–88% B; 12–15 min, 80% B isocratic, and 15–16 min, 80–85% B. A post‐run of 10 min was required for column re‐equilibration. The flow rate was set at 0.4 mL min^−1^, and the injection volume was 2.5 µL for all runs. Electrospray ionization (ESI) was conducted at 350 °C, the flow rate was 12 L min^−1^ with a nebulizer gas pressure of 45 psi, and the capillary voltage was 4000 V. The mass spectrometer was operated in negative mode, and the MS/MS data were acquired in multiple reaction monitoring (MRM) mode. MRM conditions for the analysis of the phenolic compounds studied using HPLC‐ESI‐MS/MS were conducted as previously described.^[^ ^25^ ^]^ The total polyphenol content, the individual flavanols, and the phenolic acids comprising the extract were detailed in **Table** **1**.

**TABLE 1:** Main phenolic compounds (flavanols and phenolic acids) of the grape seed proanthocyanidin extract (GSPE) used in this study, analyzed by HPLC‐MS/MS.

Phenolic compound	(M–H)‐	Calibration curve	Total amount [mg g^−1^]	SD
Protocatechuic acid (PCA)	153.0187	*y* = 1E + 06*x*	1.40	0.25
Catechin	289.0712	*y* = 1E + 06*x*	51.88	5.56
Epicatechin	289.0712	*y* = 935152*x*	62.86	8.32
Gallic acid	169.0136	*y* = 287040*x*	44.66	7.76
Kaempferol‐3‐glucoside	447.0927	*y* = 1E + 06*x*	0.50	0.02
Naringenin‐7‐glucoside	433.1135	*y* = 3E + 06*x*	0.64	0.08
p‐Coumaric acid	163.0395	*y* = 2E + 06*x*	0.09	0.01
Quercetin	301.0348	*y* = 2E + 06*x*	0.05	0.01
Quercetin‐3‐O‐galactoside	463.0877	*y* = 2E + 06*x*	0.43	0.05
Vanillic acid	167.0342	*y* = 1E + 06*x*	0.09	0.01
Procyanidin dimer	577.1346	y = 664077*x*	76.84	15.76
Procyanidin trimer	865.1979	*y* = 664077*x*	13.04	0.64
Procyanidin tetramer	1153.2613	*y* = 664077*x*	5.14	0.28
Dimer gallate	729.1455	*y* = 1E + 06*x*	15.22	2.72
Epicatechin gallate	441.0821	*y* = 1E + 06*x*	14.24	2.76
Epigallocatechin gallate	457.077	*y* = 1E + 06*x*	0.06	0.01

### 2.2 Animal Handling

All procedures involving the use and care of animals were reviewed and approved by the Animal Ethics Committee of the Universitat Rovira i Virgili (permit number 9495, September 18, 2019). All experiments were performed in accordance with relevant guidelines and regulations of the Council of the European Union and the procedure established by the Departament d'Agricultura, Ramaderia i Pesca of the Generalitat de Catalunya.

Ninety‐six 12‐week‐old male Fischer 344 rats were purchased from Charles River (Barcelona, Spain). The animals were housed in pairs in a 12‐h/12‐h light/dark cycle at 22 °C, 55% humidity, and were provided a standard chow diet (STD) and tap water ad libitum. The STD diet composition was 20% protein, 8% fat, and 72% carbohydrates (Panlab, Barcelona, Spain). After a 4‐day adaptation period, the animals were randomly divided into two groups depending on the diet; 32 rats were fed with STD, and 64 rats were fed a CAF during a 5‐week pre‐treatment. CAF consisted of biscuits with cheese and pâté, bacon, coiled puff pastry from Mallorca (Hacendado, Spain), feed, carrots, and sweetened milk (22% sucrose w/v). CAF composition was 14% protein, 35% fat, and 76% carbohydrates. After the pre‐treatment period, rats were divided into two groups according to the *Zeitgeber* time (ZT) when the treatment was administered: 48 rats were treated at the beginning of the light phase (8 a.m., ZT0), and 48 rats were treated at the beginning of the dark phase (8 p.m., ZT12). The treatment period started on the 5th week and lasted for 4 weeks. Rats continued with the diet they were fed during the pre‐treatment period. All STD‐fed rats were treated with commercial sweetened skim condensed milk (Nestle; 100 g: 8.9 g protein, 0.4 g fat, 60.5 g carbohydrates, 1175 kJ) as vehicle (VH). CAF‐fed rats were divided into two groups; 32 were treated with VH, and 32 with 25 mg kg^−1^ GSPE diluted 1/5 in condensed milk. The treatment was orally administered daily using a syringe. The rats were fasted for 3 h, then sacrificed by decapitation under anesthesia (sodium pentobarbital, 50 mg kg^−1^ per body weight). To assess diurnal transcript and metabolite profile, each diet‐treatment group was divided into four sub‐groups (*n* = 4), depending on the time of sacrifice (9 a.m. [ZT1], 3 p.m. [ZT7], 9 p.m. [ZT13], or 3 a.m. [ZT19]). Part of the liver was fixed with 10% formalin; the rest of the liver tissue was quickly frozen in liquid nitrogen and then stored at −80 °C for further analysis.

### 2.3 RNA Extraction

RNA from the liver or cells was extracted using TRIzol reagent as previously described.^[^ ^21^ ^]^ In brief, RNA was precipitated using isopropanol, washed in ethanol (70%), air‐dried, and resuspended in DNA/RNAse‐free water. RNA concentration (ng µL^−1^) and purity were measured by a Nanodrop spectrophotometer (Thermo Fisher, Germany). High‐quality RNA (260/230 and 280/260 >1.8) was used.

### 2.4 Gene Expression Analysis

Complementary deoxyribonucleic acid (cDNA) was obtained by reverse transcription of the RNA extracted using a high‐capacity complementary DNA reverse‐transcription kit (Thermo Fisher, Madrid, Spain). Quantitative polymerase chain reactions (qPCRs) were performed as previously described.^[^ ^21^ ^]^ Peptidylprolyl isomerase A (*Ppia*) and Eukaryotic translation elongation factor 1 alpha (*eEf1a*) were used as normalizers for the liver and AML‐12 cells, respectively. No marked changes in cycle thresholds (Cts) of the housekeeping genes were seen across different times. Primers used for each gene were obtained from Biomers (Ulm, Germany) and can be found in **Tables** **2** and **3**. The relative expression of each gene was calculated using the 2^−∆∆Ct^ method, as reported by Livak and Schmittgen.^[^ ^26^ ^]^

**TABLE 2:** Nucleotide sequences of primers used for real‐time quantitative PCR in liver tissue.

Gene	Forward primer (5’–3’)	Reverse primer (5’–3’)
*Acacα*	TGCAGGTATCCCCACTCTTC	TTCTGATTCCCTTCCCTCCT
*Cd36*	GTCCTGGCTGTGTTTGGA	GCTCAAAGATGGCTCCATTG
*Cyp7a1*	CACTTGTTCAAGACCGCACA	TGCTTGAGATGCCCAGAGAA
*Fasn*	TAAGCGGTCTGGAAAGCTGA	CACCAGTGTTTGTTCCTCGG
*Fatp5*	CCTGCCAAGCTTCGTGCTAAT	GCTCATGTGATAGGATGGCTGG
*G6pc*	ATTCCGGTGCTTGAATGTCG	TGGAGGCTGGCATTGTAGAT
*G6pd*	ACCAGGCATTCAAAACGCAT	CAGTCTCAGGGAAGTGTGGT
*Gk*	CTGTGAAAGCGTGTCCACTC	GCCCTCCTCTGATTCGATGA
*Ppargc1α*	AGAGTCACCAAATGACCCCAAG	TTGGCTTTATGAGGAGGAGTCG
*Pparα*	CGGCGTTGAAAACAAGGAGG	TTGGGTTCCATGATGTCGCA
*Ppia*	CCAAACACAAATGGTTCCCAGT	ATTCCTGGACCCAAAACGCT
*Sirt1*	TTGGCACCGATCCTCGAA	ACAGAAACCCCAGCTCCA
*Slc2a2*	AGTCACACCAGCACATACGA	TGGCTTTGATCCTTCCGAGT
*Srebp1c*	CCCACCCCCTTACACACC	GCCTGCGGTCTTCATTGT

**TABLE 3:** Nucleotide sequences of primers used for real‐time quantitative PCR in cells.

Gene	Forward primer (5’–3’)	Reverse primer (5’–3’)
*Acacα*	GTCCCCAGGGATGAACCAATA	GCCATGCTCAACCAAAGTAGC
*eEf1a*	TGCCCCAGGACACAGAGACTTCA	AATTCACCAACACCAGCAGCAA
*G6pc*	CGACTCGCTATCTCCAAGTGA	GTTGAACCAGTCTCCGACCA
*Gk*	AGACCTGGGAGGAACCAACT	TTTGTCTTCACGCTCCACTG
*Gys2*	GCTCTCCAGACGTTCTTGCA	GTGCGGTTCCTCTGAATGATC

### 2.5 Liver Lipid Profiling

Liver lipids were extracted following the Bligh–Dyer method.^[^ ^27^ ^]^ Levels of hepatic total cholesterol, TAG, and total lipid liver content were measured using a colorimetric kit assay (QCA, Barcelona, Spain).

### 2.6 Liver Histology

Liver portions fixed in buffered formalin (4% formaldehyde, 4 g L^−1^ NaH_2_PO_4_, 6.5 g L^−1^ Na_2_HPO_4_; pH 6.8) were cut at a thickness of 3.5 µm and stained with hematoxylin & eosin (H&E). Liver images (magnification 40×) were taken with a microscope (ECLIPSE Ti; Nikon, Tokyo, Japan) coupled to a digital camera (DS‐Ri1, Nikon) and analyzed using ImageJ NDPI software (National Institutes of Health, Bethesda, MD, USA; https://imagej.nih.gov/ij, version 1.52a).

### 2.7 Metabolomic Analysis

Metabolomic analysis of the 96 rat liver samples was performed at the Centre for Omic Sciences (COS, Tarragona, Spain) using gas chromatography coupled with quadrupole time‐of‐flight mass spectrometry (GC‐qTOF model 7200, Agilent, Santa Clara, CA, USA) as previously described.^[^ ^21^, ^28^ ^]^ Enrichment analyses were performed in the online tool MetaboAnalyst 6.0 (https://www.metaboanalyst.ca/) using the metabolite's name and KEGG pathway.

### 2.8 Alpha Mouse Liver 12 (AML‐12) *Bmal1‐luc* Maintenance

AML‐12 wild‐type cells were originally obtained from ATCC Biobank (cat number CRL‐2254) and grown in DMEM/F12 media containing 15 mM of HEPES, 1% of penicillin‐streptomycin (10 000 units mL^−1^ and 10 000 µg mL^−1^, respectively), 1% of insulin‐transferrin‐selenium (ITS), 10% of non‐heat inactivated serum (all items from Thermo Fisher, Germany). Dexamethasone at 10 nM (Sigma‐Aldirch, USA) was supplemented for regular culture maintenance. To generate *Bmal1‐luc* expressing AML‐12 cells, HEK 293 cells were transfected using CaCl_2_ solution containing *Bmal1‐luc* plasmid (17.5 µg, pABpuro‐Bluf)^[^ ^29^ ^]^ together with packing plasmids psPax and pMD2G (12.5 and 7.5 µg, respectively). The supernatant containing virus was collected 48 h later and 10‐fold concentrated using Lenti‐X Concentrator (Takara Bio, Germany). AML‐12 wild‐type cells were transduced using a concentrated virus and selected using 3 µg mL^−1^ of puromycin. From the heterogenous cell culture, single clones were identified and isolated. AML‐12 *Bmal1‐luc* clone 4 was selected and used for subsequent assays.

### 2.9 Induction of Steatosis in AML‐12 Cells

AML‐12 *Bmal1‐luc* cells were grown in the above‐mentioned media without phenol red and dexamethasone. The serum was replaced with a charcoal‐dextran‐treated serum that had a significant reduction in endogenous bovine hormones (HyClone, USA). Cells were pre‐treated with (+)‐catechin or (−)‐epicatechin 10 µM or 100 µM for 72 h, using dimethylsulfoxide (DMSO) as a solvent at 0.01% v/v or 0.1% v/v, respectively. 2 × 10^5^ cells were seeded in 35‐mm dishes containing medium with (+)‐catechin or (−)‐epicatechin at 10 µM or 100 µM. 24 h later, cells were synchronized using 200 nM of dexamethasone for 2 h. Cells were washed with PBS and loaded with media containing (+)‐catechin or (−)‐epicatechin at 10 µM or 100 µM. To mimic a steatosis scenario in vitro, cells were loaded with palmitate–bovine serum albumin (BSA) solution (6:1, 0.25 mM) or with BSA (0.04 mM) as control, both with 0.1% v/v DMSO. In all conditions, stock solutions were highly concentrated and underwent serial dilution to remove organic solvents. Stock solution of 5 mM palmitate: 0.8 mM BSA was acquired from Cayman, USA and filtered using a 0.22 µm filter. Luciferin (200 µM) was added to each dish, which was covered with a round glass lid and sealed with parafilm. All dishes were transferred to the Lumicycle and bioluminescence was measured every 10 min (Actimetrics, USA) at 32.5 °C for 4 days. Bioluminescence measurements were collected, and the raw data were imported to the Lumicycle software. Baseline subtracted bioluminescence, calculated by 24‐h running‐average subtraction, was used for rhythm evaluation.

### 2.10 Rhythm Analysis

Rhythm detection and analysis were performed using the CircaCompare algorithm.^[^ ^30^ ^]^ Rhythmic parameters were compared in a pairwise fashion using CircaCompare. The presence of rhythmicity was considered when a *p*‐value <0.05 was achieved. Comparisons of amplitudes and mesors were performed for genes or metabolites, regardless of rhythmicity status. Phase estimation was performed only on genes or metabolites that showed significant rhythms in both groups, as previously reported.^[^ ^31^ ^]^ For bioluminescence data, rhythmicity was assessed using the CircaSingle algorithm as previously described.^[^ ^32^ ^]^ CircaSingle algorithm was set to consider a dampened amplitude and period was not pre‐established. Rhythmicity was confirmed when *p*‐value <0.05.

### 2.11 Statistical Analysis

All data, except for bioluminescence data, were log2 transformed. Hepatic lipid, metabolite, and gene expression oscillations were analyzed as described above. Principal Component Analyses (PCA) were performed using the factoextra package and Hartigan‐Wong, Lloyd, and Forgy MacQueen algorithms (version 1.0.7) in R. Liver lipid profiles were subjected to Student's *t*‐test and one‐way ANOVA followed by Turkey's multiple‐range post‐hoc test using IBM SPSS Statistics (version 25) software. Graphics were done in GraphPad Prism 9 software (San Diego, CA, USA) and R using ggplot2 package. For all analyses, a *p*‐value of <0.05 was used to reject the null hypothesis.

## 3 Results

### 3.1 Evening GSPE Administration Is More Beneficial than Morning Treatment

**FIGURE 1:**
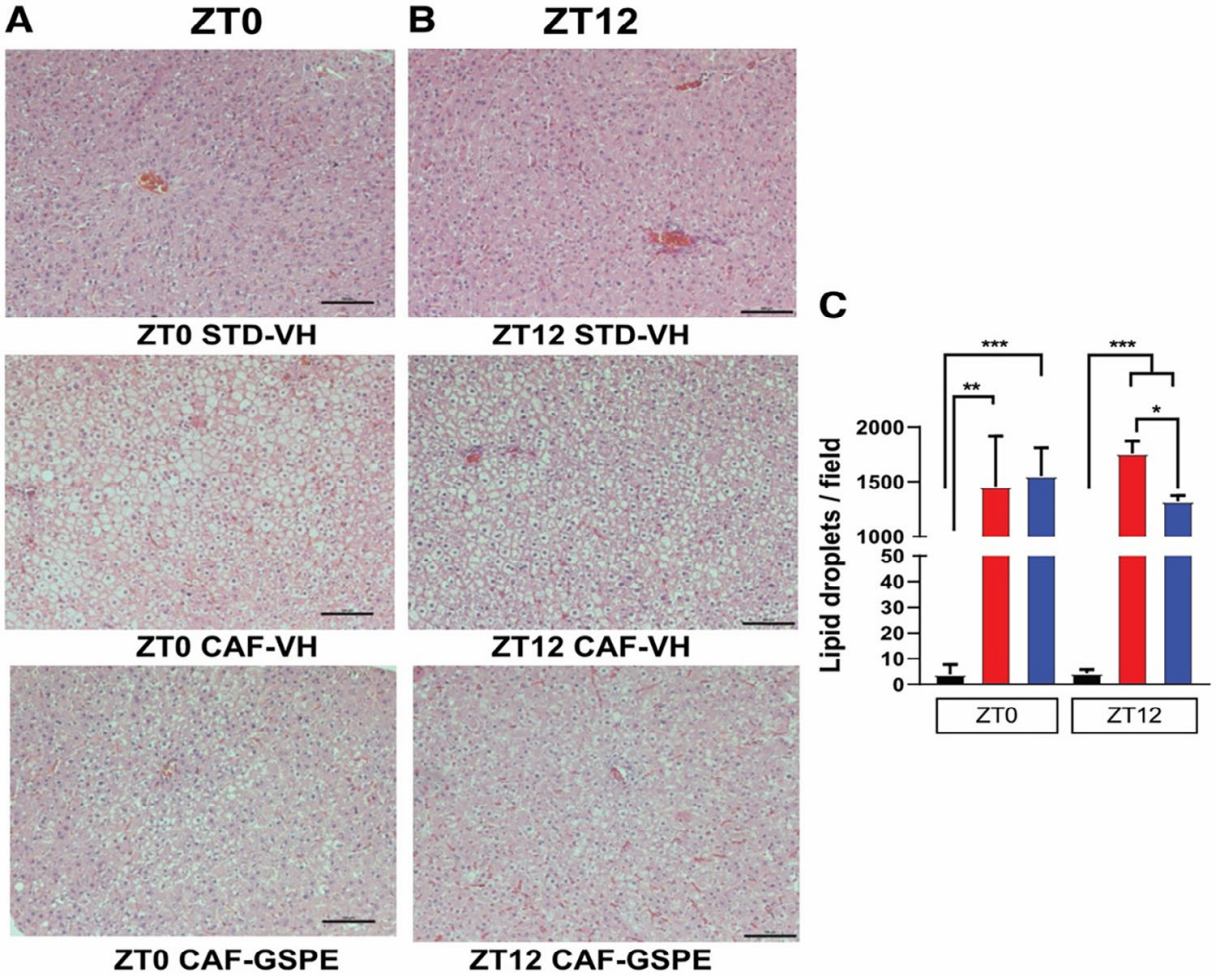
Representative pictures of H&E‐stained liver sections. Scale bar, 100 µm. Rats were fed a STD or CAF diet and received a daily dosage of vehicle or GSPE at the beginning of the light phase (ZT0) (A) or at the beginning of the dark phase (ZT12) (B). Quantitative analysis of lipid droplets in liver samples per microscopic field (100×) (C). * Indicates significant differences (Student's *t*‐test, *p < 0.05, **p < 0.01, ***p < 0.001). Shown are means ± SEM (*n* = 16/group).

Our experimental design consisted of inducing liver steatosis by feeding rats a CAF diet for 5 weeks. Additional GSPE treatment at the beginning of the inactive (morning, ZT 0) or beginning of the active phase (evening, ZT 12) was given daily for additional 4 weeks. CAF‐VH led to marked liver lipid accumulation compared to the control group. GSPE treatment in the evening—but not during the morning—reduced hepatic lipid content in CAF‐fed rats (**Figure** **1**A–C). Interestingly, as it has been previously described, despite any major significant biochemical change in the main plasmatic parameters being observed in these animals, a significant weight loss was evidenced only in the ZT12 condition without any change in food intake among CAF diet experimental groups.^[^ ^33^ ^]^ Importantly, in the present research, the biochemical lipid profile of the livers when the animals are clustered in ZT0 and Z12 groups (Figure S1, Supporting Information), evidences a greater impact of the GSPE intervention at ZT12, as can be seen in the significant decrease of liver triglycerides in this condition (Figure S1B, Supporting Information) as well as the in the absence of a significant increase, compared with the STD animals, in cholesterol (Figure S1A, Supporting Information) and total lipids (Figure S1C, Supporting Information) which was paradoxically present in the ZT0 condition. Altogether, these results confirm that only the ZT12‐treated group showed a significant protection against CAF‐induced metabolic changes and reinforce the importance of timing in GSPE's metabolic effects.

**FIGURE 2:**
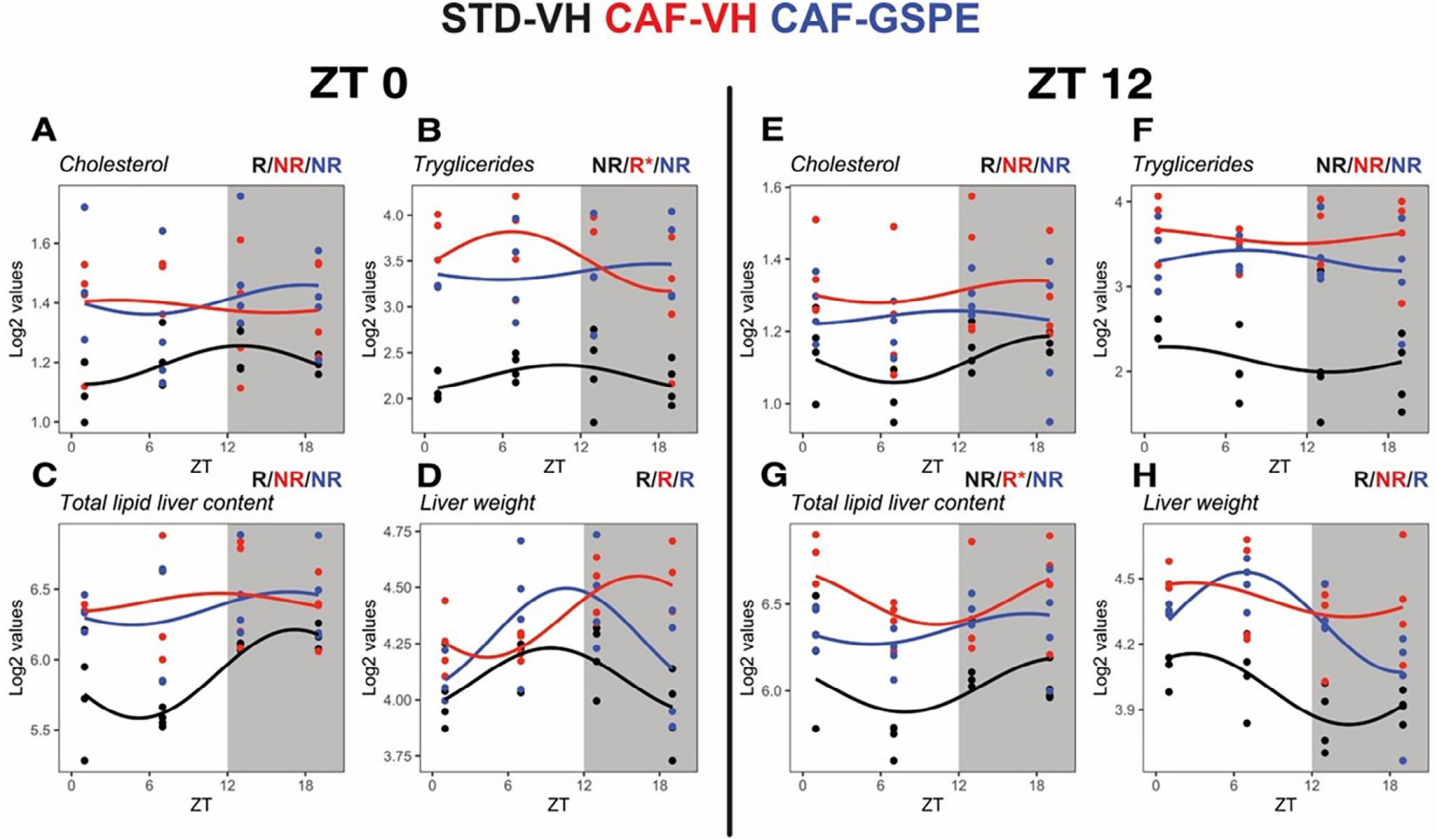
Diurnal rhythms of liver lipid levels and liver weight. Rats were fed STD or CAF diet and received a daily dosage of vehicle or GSPE at the beginning of the light phase (ZT0) (A–D) or at the beginning of the dark phase (ZT12) (E–H). Rhythm parameter determination and comparison were performed using CircaCompare. R/NR indicates significant/non‐significant rhythmicity (*p* < 0.05). R* shows a tendency of rhythmicity (*p* < 0.1). *N* = 3 – 4/group at each time point.

Diurnal profile analysis showed that cholesterol levels were rhythmic in STD‐VH animals and arrhythmic after CAF diet, without any effect in the GSPE‐treated group. Triglycerides only showed a tendency to rhythmicity in the CAF‐VH group and GSPE did not affect any of the rhythm parameters. Diurnal oscillations in liver weight were observed in STD‐VH‐treated animals. In the inactive phase‐treated group, CAF‐VH phase delayed the peak of liver weight by 7 h, which was reverted by GSPE treatment. In the evening treatment group, CAF led to the loss of rhythmicity in liver weight, which GSPE also reverted with a tendency of amplitude difference (*p* = 0.09, **Figure** **2**A–H; Table S1, Supporting Information).

### 3.2 Differential Effects of CAF and GSPE on Diurnal Lipid Metabolism

**FIGURE 3:**
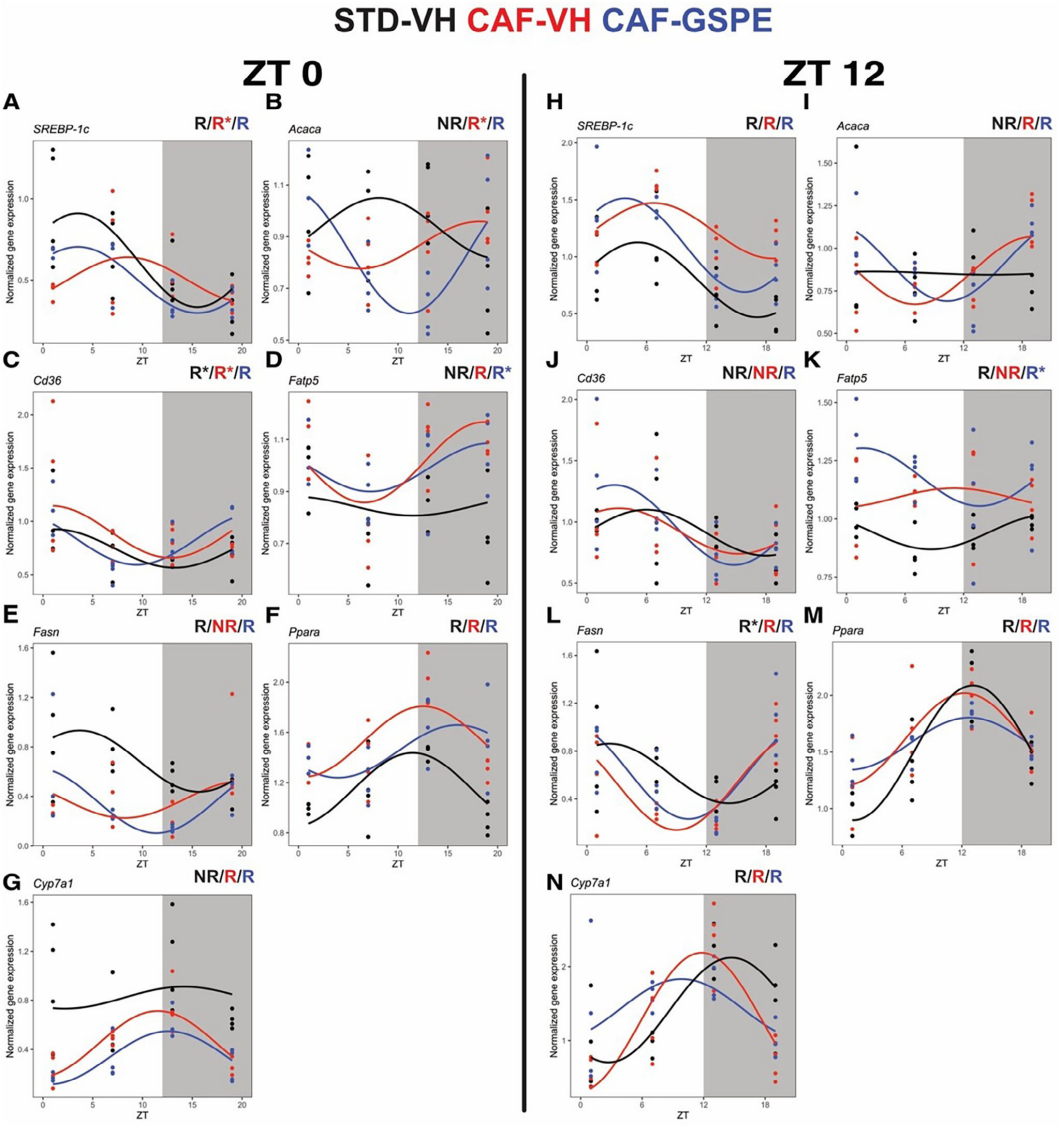
Diurnal gene expression profiles of key genes involved in lipid and bile acid metabolism. Rats were fed a STD or CAF diet and received a daily dosage of vehicle or GSPE at the rest phase (ZT0) (A–G) or at the resting phase (ZT12) (H–N). Rhythm parameter determination and comparison were performed using CircaCompare. R/NR indicates significant/non‐significant rhythmicity (*p* < 0.05). R* shows a tendency of rhythmicity (*p* < 0.1). *N* = 3–4/group at each time point.

For rats treated at the inactive phase, the gene expression of STD‐VH group was rhythmic for sterol regulatory element‐binding protein 1c (*SREBP‐1c*), *Cd36*, fatty acid synthase (*Fasn*), and peroxisome proliferator‐activated receptor α (*Pparα*). After CAF, rhythmicity was lost only for the expression of *Fasn*, which was restored under GSPE treatment, albeit without significant changes in amplitude. Additionally, acetyl‐CoA carboxylase alpha (*Acaca)*, fatty acid transport protein‐5 (*Fatp5*), and cholesterol 7α‐hydroxylase (*Cyp7a1*) gained rhythmicity following the CAF regimen without any significant changes in amplitude (**Figure** **3**A–G; Table S2, Supporting Information).

For rats treated during the active phase, rhythmic expression was identified for *SREBP‐1c*, *Fatp5*, *Fasn*, *Ppara*, and *Cyp7a1*. In response to CAF, rhythmicity was lost only *Fatp5* without any amplitude differences in addition to a mesor increase compared to STD‐VH. In addition, *Fasn* showed a borderline rhythmicity (*p* = 0.053) in STD, which was phase‐advanced compared to CAF‐VH and CAF‐GSPE groups. In CAF rats, *Cyp7a1* showed a phase advance of 3 h compared to the STD group. Interestingly, *Acaca* expression was arrhythmic in STD, while GSPE‐treated rats led to a phase delay of 3 h compared to CAF‐VH group. (Figure 3H–N; Table S2, Supporting Information).

### 3.3 Differential Effects of CAF and GSPE in the Diurnal Glucose Metabolism Expression

**FIGURE 4:**
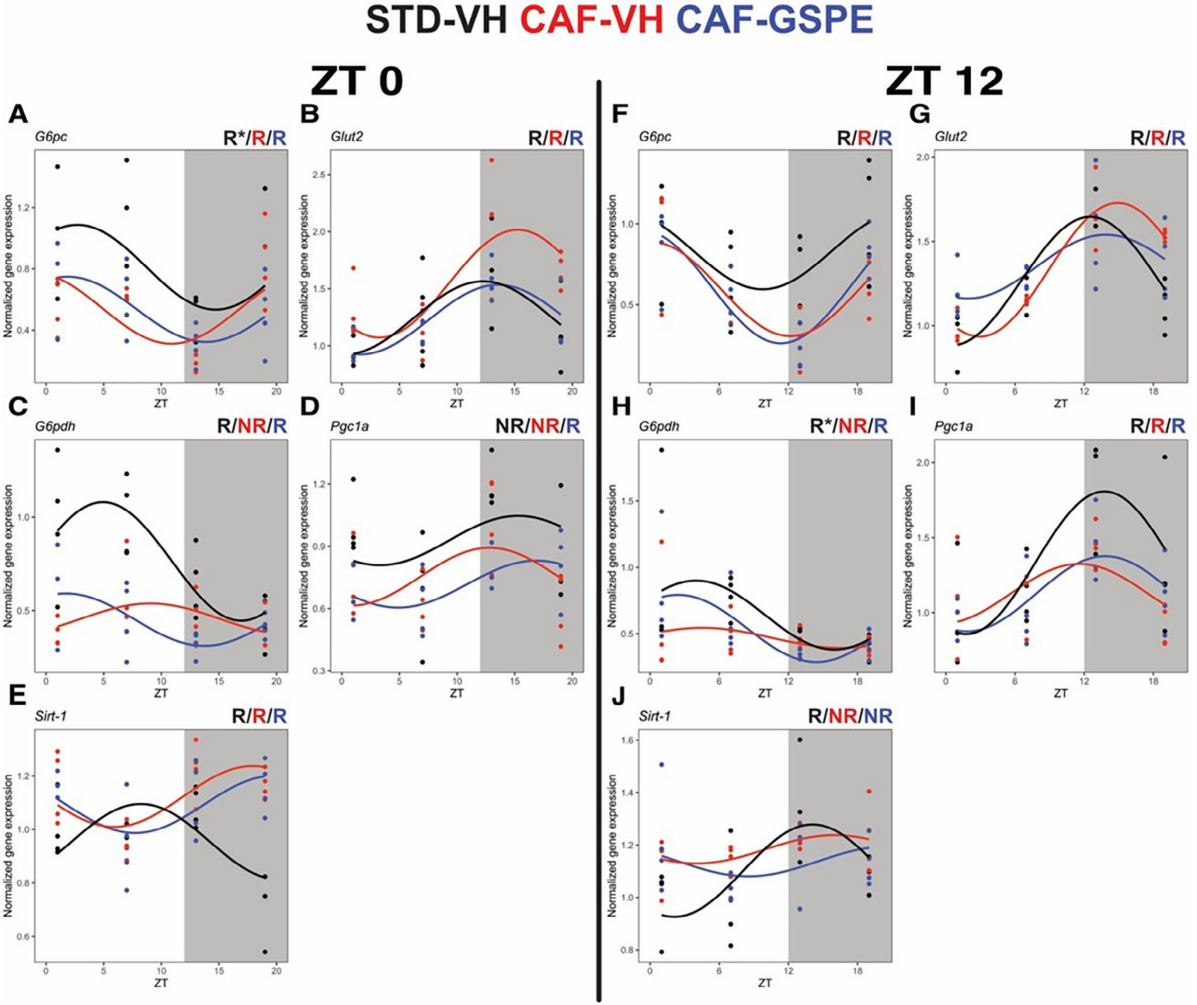
Diurnal gene expression profiles of key genes glucose metabolism. Rats were fed a STD or CAF diet and received a daily dosage of vehicle or GSPE at the rest phase (ZT0) (A–G) or at the resting phase (ZT12) (H–N). Rhythm parameter determination and comparison were performed using CircaCompare. R/NR indicates significant/non‐significant rhythmicity (*p* < 0.05). R* shows a tendency of rhythmicity (*p* < 0.1). *N* = 4/group at each time point.

In the group treated in the resting phase, rhythmicity was detected for *G6* *pc*, *Glut2*, *G6pdh*, and *Sirt1*. CAF‐VH reduced the mesor on glucose‐6‐phosphatase (*G6* *pc*) and glucose‐6‐phosphate dehydrogenase (*G6pdh*). Reduced amplitude on *G6pdh* resulted in arrhythmicity in CAF‐VH compared to the STD group treated in the resting phase. Treatment with GSPE restored the rhythmicity of *G6pdh*, although without significant amplitude change. Increased mesor was identified for glucose transporter 2 (*Glut2*) and sirtuin 1 (*Sirt1*) in response to CAF. GSPE treatment led to reduced *Glut2* mesor compared to CAF‐VH, like STD‐VH values. CAF‐VH led to a phase delay on *Sirt1* expression of approximately 9 h, which GSPE treatment did not revert (**Figure** **4**A–E; Table S2, Supporting Information).

The presence of rhythmicity was identified in all genes in the evening‐treated group. The effects of the CAF‐VH diet in the group treated in the evening led to a decreased *G6* *pc* expression and a 2 h phase delay in *Glut2* compared to the STD‐VH group, which was unaffected by GSPE treatment. Notably, GSPE treatment reduced *Glut2* amplitude compared to CAF‐VH group. CAF‐VH resulted in *Gp6dh* arrhythmicity, which was restored by GSPE treatment, albeit without amplitude changes (*p* = 0.14). *Sirt1* expression was rhythmic in the STD‐VH group and lost in CAF‐VH and CAF‐GSPE groups, albeit without amplitude parameter changes (Figure 4F–J; Table S2, Supporting Information).

### 3.4 Impact of CAF and GSPE on the Rhythmicity of Liver Metabolites

To understand the effects of an obesogenic diet on liver metabolism, we conducted a comprehensive metabolomic analysis. This analysis identified and quantified 61 metabolites across all groups in our study. Using PCA we noted distinctions in liver metabolite profiles between groups treated at ZT0 and ZT12, indicative of a time‐of‐day influence of GSPE treatment (Figure S2, Supporting Information).

In rats treated in the morning phase, mesor evaluation, regardless of the presence of rhythmicity, identified six (e.g., nicotinamide, myo‐inositol, cholesterol, pyruvic acid, sarcosine, and urea) and seven metabolites (e.g., threonine, glutamic acid, ribose 5‐phosphate, serine, inosine 5‐monophosphate, oleic acid‐iso1, d‐ribose) as down‐ or upregulated in response to CAF‐VH, respectively. Overall, enrichment analysis identified that the metabolites affected by CAF‐VH were associated with glycine, serine, threonine, glyoxylate, and ascorbate processes. GSPE treatment, in comparison to CAF‐VH, reduced the levels of four metabolites (e.g., glucose 6‐phosphate, fructose 6‐phosphate, oxoproline, taurine) and increased the levels of additional four metabolites (a‐ketoglutaric acid, fumaric acid, sarcosine, d‐glucuronic acid). These metabolites are enriched for arginine biosynthesis and citrate cycle (Table S3, Supporting Information).

On the other hand, in rats treated in the active phase, CAF‐VH reduced 16 metabolites (e.g., nicotinamide, methionine, sarcosine, isoleucine, etc.), but it increased only two metabolites (serine and oleic acid‐iso1). These metabolites were mostly associated with valine, leucine, isoleucine ascorbate, threonine, cysteine, and methionine metabolism. Intriguingly, GSPE treatment resulted only in the upregulation of serine compared to CAF‐VH (Table S3, Supporting Information).

**FIGURE 5:**
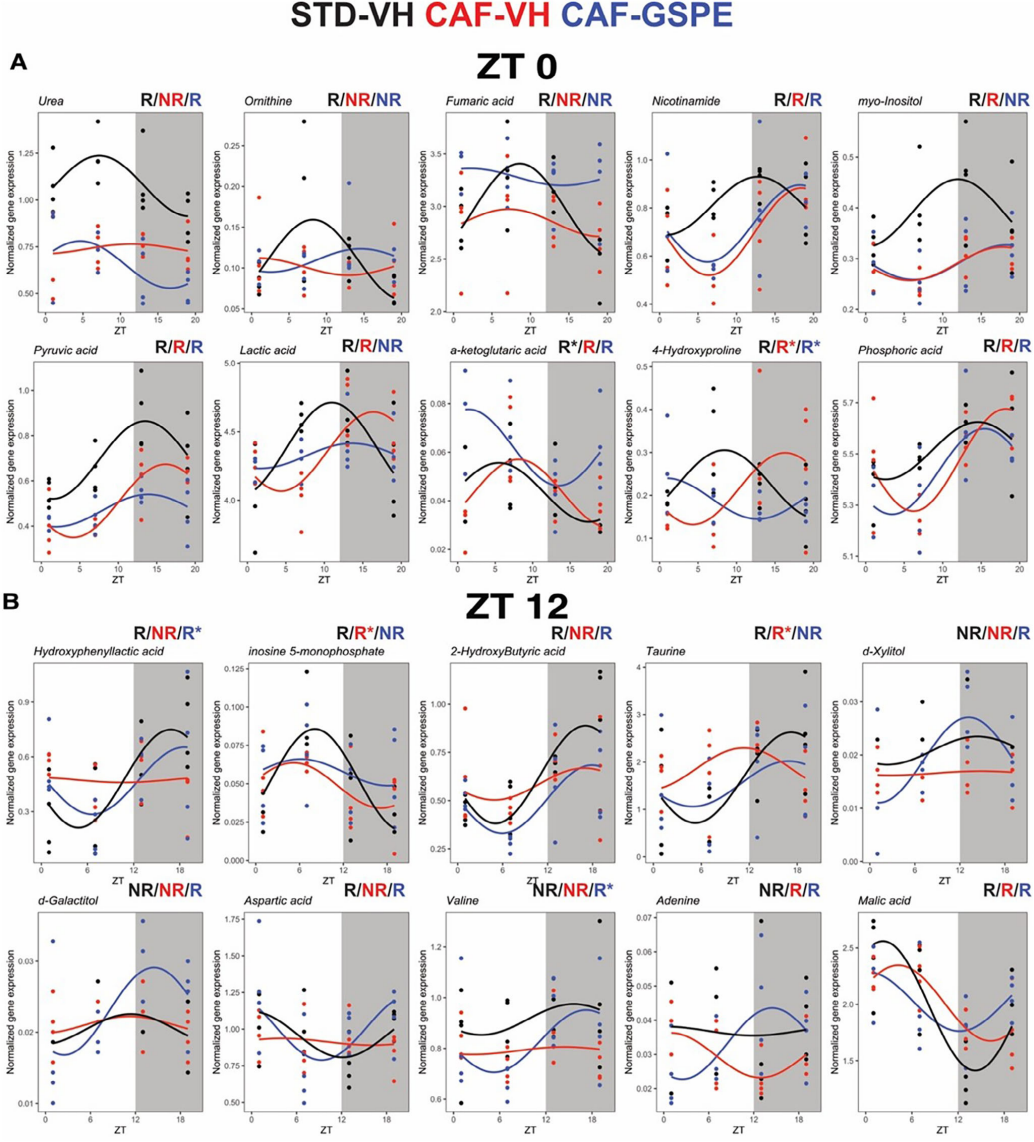
Diurnal hepatic metabolites affected by CAF diet and GSPE treatment given during the inactive or active phase (A–B). Selected metabolites with rhythm parameter alterations in rats treated during the rest or active phase. Rhythm parameter determination and comparison were performed using CircaCompare. R/NR indicates significant/non‐significant rhythmicity (p < 0.05). R* shows a tendency of rhythmicity (p < 0.1). Shown are means ± SEM (*n* = 4/group at each time point).

Rhythmic analysis of hepatic metabolites focused on amplitude and phase changes induced by CAF and affected by GSPE revealed interesting findings. In the group treated during the light phase, 29, 33, and 27 rhythmic metabolites were identified in STD‐VH, CAF‐VH, and CAF‐GSPE, respectively (Table S3, Supporting Information). Differential rhythm analyses identified that the CAF‐VH reduced the amplitude, which resulted in the loss of rhythmicity, with a tendency of reduced amplitude of urea (*p* = 0.07), ornithine (*p* = 0.08), and fumaric acid (*p* = 0.1). Conversely, CAF‐VH phase delayed nicotinamide (ca. 6 h), myo‐inositol (ca. 6 h), sedoheptulose (ca. 9 h), alanine (ca. 8 h), d‐Galactitol (ca. 6 h), lactic acid (ca. 5 h), and proline (ca. 5 h). GSPE‐treated at the inactive phase tended to reduce amplitude (without rhythm loss) of pyruvic acid (*p* = 0.06) and loss of rhythmicity of lactic acid (*p* = 0.08). GSPE treatment phase advanced the phase of a‐ketoglutaric acid (ca. 7 h) and phosphoric acid (*p* = 0.07, ca. 3 h) while it delayed 4‐hydroxyproline phase by ca. 8 h in comparison to the CAF‐VH group (**Figure** **5**A; Table S3, Supporting Information).

On the other hand, CAF‐VH effects in the active phase‐treated group had less rhythmic metabolites. A total of 23, 14, and 12 metabolites were identified in the STD‐VH, CAF‐VH, and CAF‐GSPE groups, respectively (Table S3, Supporting Information). CAF‐VH effects on liver diurnal metabolome resulted in amplitude reduction that led to rhythmicity loss of hydroxyphenyllactic acid, inosine 5‐monophosphate (*p* = 0.09), 2‐hydroxybutyric acid (*p* = 0.1), and phase advance of taurine. Conversely, GSPE treatment at the active phase resulted in gain rhythmicity with a tendency of significant amplitude change of d‐xylitol, d‐galactitol (*p* = 0.06), aspartic acid (*p* = 0.09), and valine (*p* = 0.14) while it shifted the phase of adenine by 10 h and advanced malic acid peak (ca. 5 h) in comparison to CAF‐VH group (Figure 5B; Table S3, Supporting Information).

### 3.5 (+)‐Catechin Restores Circadian Rhythm and Metabolic Gene Expression in an In Vitro Model of MASLD

**FIGURE 6:**
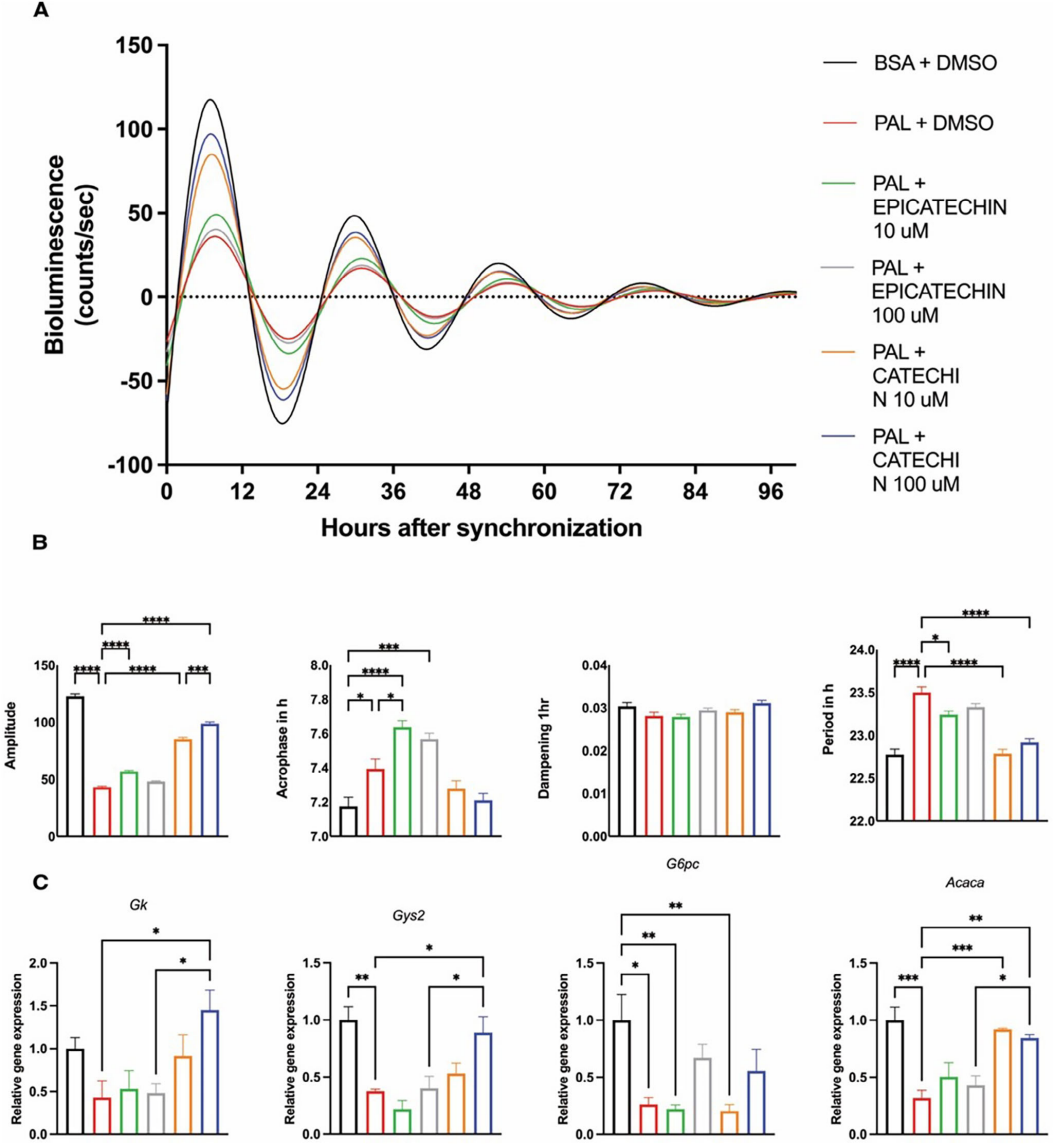
Effects of palmitate/catechin or epicatechin treatment on circadian clock rhythms in hepatocytes in vitro. A) Luminescence rhythms in synchronized AML12 hepatocytes stably expressing Bmal1‐luc reporter and after treatment with GSPE. Representative dampened sine curve fits on normalized bioluminescence data are shown. B) Circadian rhythm parameters of AML12 Bmal1‐luc cells (amplitude, acrophase, period, and dampening) in response to palmitate/GSPE treatment. C) Gene expression levels of metabolic genes in AML12/Bmal1‐luc cells treated with catechin or epicatechin (10 µM or 100 µM) for 96 h and cultured with bovine serum albumin (BSA) conjugated with palmitate (PAL) (0.25 mM) with 0.1% v/v DMSO. Cells cultured in BSA (0.04 mM) with 0.1% v/v DMSO were used as control. Data are means ± SEM (*n* = 4). One‐way ANOVA followed by Tukey's post‐test was performed to compare values between groups (*p < 0.05, **p < 0.01, ***p < 0.001).

HPLC analysis revealed that (+)‐catechin and (−)‐epicatechin are key phenolic compounds in GSPE (Table 1). Considering the effects identified in vivo, we sought to evaluate the effect of (+)‐catechin and (−)‐epicatechin in an in vitro hepatocyte model. We used a *Bmal1‐luc* reporter line that allows the real‐time measurement of *Bmal1* promoter activity. Cells were treated with palmitate to simulate hepatic steatosis and then exposed to different concentrations of catechin or epicatechin. Incubation with palmitate resulted in amplitude reduction and increased period. Interestingly, only (+)‐catechin effectively restored circadian rhythm disturbances caused by palmitate, including amplitude, phase, and period. Additionally, (+)‐catechin restored the expression of key metabolic genes such as *Gk*, *Gys2*, and *Acaca*, reversing the effects of palmitate to control levels (**Figure** **6**A–L). The quantification data on the amplitude, phase, and period of the rhythms upon all conditions in the in vitro model as well as the statistical data on the condition comparisons have been included in the manuscript as a supplemental table (Table S4, Supporting Information).

## 4 Discussion

Our study shows that GSPE treatment at the active (dark) phase (ZT 12) significantly reduced hepatic lipid content compared to rats treated at their inactive (light) phase (ZT 0). These results align with previous research that identified that GSPE treatment in CAF‐fed rats in the evening reduces body weight, while such an effect is absent in rats treated in the light phase.^[^ ^21^ ^]^ This finding underscores the importance of biological rhythms in therapeutic strategies for MASLD, where lipid metabolism is intrinsically linked to the liver clock.^[^ ^5^ ^]^ Moreover, we have previously identified the beneficial effects of GSPE treatment at the active phase in restoring the CAF‐induced clock gene (e.g., *Bmal1*, *Per2*, *Cry1*, *Rorα*) disruption in the liver.^[^ ^21^ ^]^ This restoration likely facilitated the normalization of downstream metabolic processes, as evidenced by the reestablishment of rhythmic expression of lipogenic and lipid transport‐related genes (e.g., *Acaca* and *Fatp5*) and their metabolic outputs. Interestingly, our findings further suggest that GSPE modulates diurnal liver mass rhythms, highlighting it as a strong modulator of liver physiological processes. The phase shifts and loss of rhythmicity induced by the CAF‐VH were effectively counteracted by GSPE, with the most pronounced effects observed with nocturnal supplementation as GSPE rhythmicity in liver mass that was disrupted by the CAF diet. In this regard, previous studies have shown that polyphenols like resveratrol exhibit time‐dependent effects. For instance, resveratrol administered during the dark phase acted as an antioxidant, while during the light phase, it had pro‐oxidant effects.^[^ ^34^ ^]^ Additionally, a study on proanthocyanidins showed that administration time affected the expression of clock genes and serum metabolite levels in a sex‐ and diet‐dependent manner.^[^ ^35^ ^]^ This suggests that the timing of polyphenol administration could significantly affect their biological action.

The diurnal metabolic analysis allowed a comprehensive view of metabolite dynamics across conditions and time. In both groups, CAF led to a marked alteration in rhythm parameters, i.e., amplitude and phase in metabolites. Overall, changes induced by CAF did not overlap and, thus, were associated mostly with a particular group (e.g., ZT0 or ZT12). Importantly, GSPE treatment led to marked effects in both treatment groups. Our study identified several metabolites that were significantly different in the CAF‐VH compared to the STD‐VH. One of the affected metabolites was urea. Several studies demonstrated an association between liver injury in MASLD and downregulation of the urea cycle components, which alter the functional capacity for ureagenesis.^[^ ^36^, ^37^ ^]^ Our results showed not only a decrease in urea levels in animals fed a CAF diet but also a loss of rhythmicity in hepatic urea and in some urea cycle intermediate metabolites (ornithine and aspartic acid). This correlates with previous observations that describe such alteration in urea cycle metabolites as a sensor of hepatocyte mitochondrial damage.^[^ ^38^ ^]^ In this regard, our results showed that treatment with GSPE restored the diurnal oscillation of urea at ZT0 and of aspartic acid at ZT12, albeit without amplitude changes. Another reported effect of CAF‐VH, which can affect liver homeostasis, is the rhythmicity loss of TCA cycle intermediates, such as pyruvic, succinic, fumaric, malic, a‐ketoglutaric, and citric acids.^[^ ^21^ ^]^ Thus, considering the key role of the mitochondria in energy metabolism, the partial restoration of TCA cycle diurnal rhythmicity by GSPE supplementation suggests beneficial effects on hepatic metabolic homeostasis.

In addition, 2‐hydroxybutyric acid has been reported as an early biomarker of insulin resistance and impaired glucose balance^[^ ^39^ ^]^ and recently was identified as an exercise‐induced metabolite that may reflect the cytosolic redox state and energy stress at a systemic level.^[^ ^40^ ^]^ CAF‐VH led to 2‐hydroxybutyric rhythm loss, which was restored in GSPE in the active phase. Moreover, GSPE treatment at the inactive phase modestly reduced the amplitude of pyruvic acid and led to the loss of rhythmicity in lactic acid, with phase shifts in a‐ketoglutaric and phosphoric acids. Conversely, in the active phase‐treated group, the GSPE treatment increased amplitude and rhythmicity for metabolites such as d‐xylitol and aspartic acid and altered the phase of adenine and malic acid. Collectively, these findings show the potential of GSPE to re‐establish disrupted metabolic rhythms induced by a CAF diet.

Concerning inflammation, other experiments with longer CAF diet treatments (14 weeks) than the used in the present research (9 weeks) have shown that the improvement of the metabolic state of the obese rat due to GSPE's interventions is correlated with a decrease in systemic inflammation.^[^ ^41^ ^]^ Consequently, it will be an interesting future perspective to also study the interaction of inflammation and CAF‐related circadian disruption in our experimental model.

On the other hand, from the different compounds present in the GSPE extract, we provide evidence for the strong role of (+)‐catechin, which was able to rescue—in a dose‐dependent manner—the dampened *Bmal1‐luc* rhythms in an in vitro steatosis model of murine hepatocytes. In addition to modulating the core clock, (+)‐catechin rescued the expression of key other metabolic genes, such as *Gk*, *Gys2*, and *Acaca*, which were disrupted by steatosis. Accordingly, in vitro antioxidant and anti‐inflammatory activities of (+)‐catechin treatment have been evidenced in hepatocytes. The addition of such compound modulated gene expression of *Nrf2* and *NF‐kB* inflammatory pathways, and regulated key enzymes involved in oxidative stress, thus leading to potential hepatoprotective effects.^[^ ^42^, ^43^, ^44^ ^]^ Moreover, (+)‐catechin inhibited 3T3‐L1 preadipocyte differentiation via modulating the C/EBP/PPARγ/SREBP‐1c pathway and stimulated lipid degradation of mature adipocytes through cAMP/PKA pathways.^[^ ^45^ ^]^

Importantly, concerning the translation to humans, in the present experiment, GSPE was administered at 25 mg kg^−1^ of body weight to rats. According to the Reagan–Shaw conversion method,^[^ ^46^ ^]^ this dose translates to approximately 284 mg day^−1^ for a 70 kg human. While this intake could theoretically be achieved through a polyphenol‐rich diet, it would require careful dietary planning, as proanthocyanidins are typically found in foods like grapes, berries, and red wine.

Regarding the in vitro model, several studies^[^ ^47^, ^48^ ^]^ have confirmed that palmitate is a reliable model for exploring the cellular processes involved in diet‐induced obesity. However, lab conditions differ from the complex interactions that occur in a living system, and this limitation has to be taken into account. Importantly, our metabolomics analyses do not reflect the entire liver metabolome due to the extraction method used. Finally, the fact that GSPE restored circadian rhythms regardless of the administration time, while only reducing liver lipids at ZT12, suggests that the metabolic improvements are not solely mediated by the restoration of rhythmicity. While circadian rhythms play a role in metabolic regulation, other mechanisms—such as direct effects on lipid metabolism and gene expression—may also be involved in GSPE's protective effects at ZT12. This observation warrants further investigation. One way to clarify this would be through the use of clock‐knockout models, where it could be assessed whether the absence of circadian regulation abolishes GSPE's effects on metabolism.

In summary, our findings demonstrate that the timing of GSPE administration plays a crucial role in its efficacy in preventing and ameliorating steatosis. The synchronization of GSPE treatment with the liver's circadian rhythm not only enhances its metabolic benefits but also underscores the importance of considering circadian biology in the development of dietary interventions. By restoring the rhythmic expression of key metabolic and circadian genes, GSPE contributes to normalizing lipid and glucose metabolism, offering a promising therapeutic avenue for managing MASLD and related metabolic disorders. Our findings suggest a potential role of (+)‐catechin as one of the main bioactive compounds in the GSPE extract. Future studies are needed to explore further the mechanisms underlying the circadian modulation of GSPE effects (e.g., inflammation assessment, other solvents for hepatic metabolite extraction, as well as animal activity and feeding rhythms) and to validate these findings in clinical settings. Furthermore, although our in vitro concentrations were chosen to ensure measurable effects on circadian genes and lipid metabolism, we recognize the need to establish more accurate the in vivo concentrations in liver tissue.

## Conflict of Interest

The authors declare no conflict of interest.

## Author Contributions

R.M.R. and L.V.M.A. contributed equally to this work. R.M.R.; M.M.; G.A.; E.C.; H.O.; L.V.M.A.: conceptualization, methodology, investigation, formal analysis, visualization, writing – original draft, writing – review & editing, funding acquisition, project administration. R.M.R.; E.C.; L.V.M.A.; G.A.X.E.: investigation, formal analysis, validation, data curation. Methodology, investigation: M.C‐P.; A.C.‐C.; S.Q.‐V.; M.M; H.O.: supervision, resources.

## Supporting information



Supporting Information

Supporting Information

Supporting Information

Supporting Information

Supporting Information

## Data Availability

The data that support the findings of this study are available from the corresponding author upon reasonable request.

## References

[1] S. L. Friedman , B. A. Neuschwander‐Tetri , M. Rinella , A. J. Sanyal , Nat. Med. 2018, 24, 908. 29967350 10.1038/s41591-018-0104-9 PMC6553468

[2] K. Pafili , M. Roden , Mol. Metab. 2021, 50, 101122. 33220492 10.1016/j.molmet.2020.101122 PMC8324683

[3] E. Maury , K. M. Ramsey , J. Bass , Circ. Res. 2010, 106, 447. 20167942 10.1161/CIRCRESAHA.109.208355 PMC2837358

[4] S. R. G. Ferreira , Y. Macotela , L. A. Velloso , M. A. Mori , Nat. Metab. 2024, 6, 409. 38438626 10.1038/s42255-024-00977-1

[5] L. V. M. de Assis , M. Demir , H. Oster , Acta Physiol. 2023, 237, e13915. 10.1111/apha.13915 36599410

[6] H. Reinke , G. Asher , Gastroenterology 2016, 150, 574. 26657326 10.1053/j.gastro.2015.11.043

[7] L. V. M. de Assis , H. Oster , Cell. Mol. Life Sci. 2021, 78, 4563. 33683376 10.1007/s00018-021-03800-2 PMC8195959

[8] G. Asher , P. Sassone‐Corsi , Cell 2015, 161, 84. 25815987 10.1016/j.cell.2015.03.015

[9] K. L. Eckel‐Mahan , V. R. Patel , S. de Mateo , R. Orozco‐Solis , N. J. Ceglia , S. Sahar , S. A. Dilag‐Penilla , K. A. Dyar , P. Baldi , P. Sassone‐Corsi , Cell 2013, 155, 1464. 24360271 10.1016/j.cell.2013.11.034 PMC4573395

[10] L. V. M. de Assis , M. Demir , H. Oster , Cell. Mol. Gastroenterol. Hepatol. 2023, 16, 341. 37270062 10.1016/j.jcmgh.2023.05.008 PMC10444956

[11] L. Gu , M. A. Kelm , J. F. Hammerstone , G. Beecher , J. Holden , D. Haytowitz , R. L. Prior , J. Agric. Food Chem. 2003, 51, 7513. 14640607 10.1021/jf034815d

[12] L. Gu , M. A. Kelm , J. F. Hammerstone , G. Beecher , J. Holden , D. Haytowitz , S. Gebhardt , R. L. Prior , J. Nutr. 2004, 134, 613. 14988456 10.1093/jn/134.3.613

[13] Z. Y. Chen , P. T. Chan , K. Y. Ho , K. P. Fung , J. Wang , Chem. Phys. Lipids 1996, 79, 157. 8640902 10.1016/0009-3084(96)02523-6

[14] R. de la Iglesia , F. I. Milagro , J. Campión , N. Boqué , J. A. Martínez , BioFactors 2010, 36, 159. 20232344 10.1002/biof.79

[15] J. Yang , Y.‐Y. Xiao , Crit. Rev. Food Sci. Nutr. 2013, 53, 1202. 24007424 10.1080/10408398.2012.692408

[16] M. Quiñones , L. Guerrero , M. Suarez , Z. Pons , A. Aleixandre , L. Arola , B. Muguerza , Int. Food Res. 2013, 51, 587.

[17] M.‐L. Ricketts , B. S. Ferguson , Curr. Pharm. Des. 2018, 24, 158. 29189132 10.2174/1381612824666171129204054

[18] L. Baselga‐Escudero , A. Arola‐Arnal , A. Pascual‐Serrano , A. Ribas‐Latre , E. Casanova , M.‐J. Salvadó , L. Arola , C. Blade , PLoS One 2013, 8, e69817. 23922812 10.1371/journal.pone.0069817 PMC3724906

[19] L. Baselga‐Escudero , A. Pascual‐Serrano , A. Ribas‐Latre , E. Casanova , M. J. Salvadó , L. Arola , A. Arola‐Arnal , C. Bladé , Nutr. Res. 2015, 35, 337. 25769350 10.1016/j.nutres.2015.02.008

[20] J. Miranda , M. P. Portillo , J. A. Madrid , N. Arias , M. T. Macarulla , M. Garaulet , Br. J. Nutr. 2013, 110, 1421. 23537522 10.1017/S0007114513000755

[21] R. M. Rodríguez , A. J. Cortés‐Espinar , J. R. Soliz‐Rueda , C. Feillet‐Coudray , F. Casas , M. Colom‐Pellicer , G. Aragonès , J. Avila‐Román , B. Muguerza , M. Mulero , M. J. Salvadó , Nutrients 2022, 14, 774. 35215423 10.3390/nu14040774 PMC8876123

[22] Y. Zhang , L. Cheng , Y. Liu , R. Zhang , Z. Wu , K. Cheng , X. Zhang , J. Agric. Food Chem. 2022, 70, 1890. 35112849 10.1021/acs.jafc.1c07594

[23] Y. Cui , Y. Yin , S. Li , Y. Xie , Z. Wu , H. Yang , Q. Qian , X. Li , J. Funct. Foods 2022, 92, 105051.

[24] M. Colom‐Pellicer , R. M. Rodríguez , J. R. Soliz‐Rueda , L. V. M. de Assis , È. Navarro‐Masip , S. Quesada‐Vázquez , X. Escoté , H. Oster , M. Mulero , G. Aragonès , Nutrients 2022, 14, 2246. 35684049 10.3390/nu14112246 PMC9182881

[25] M. Margalef , L. Iglesias‐Carres , Z. Pons , F. I. Bravo , B. Muguerza , A. Arola‐Arnal , J. Nutr. Biochem. 2016, 29, 90. 26895669 10.1016/j.jnutbio.2015.11.007

[26] K. J. Livak , T. D. Schmittgen , Methods (San Diego, Calif.) 2001, 25, 402. 11846609 10.1006/meth.2001.1262

[27] E. G. Bligh , W. J. Dyer , Can. J. Biochem. Physiol. 1959, 37, 911. 13671378 10.1139/o59-099

[28] T. Cajka , O. Fiehn , Anal. Chem. 2016, 88, 524. 26637011 10.1021/acs.analchem.5b04491

[29] S. A. Brown , F. Fleury‐Olela , E. Nagoshi , C. Hauser , C. Juge , C. A. Meier , R. Chicheportiche , J.‐M. Dayer , U. Albrecht , U. Schibler , PLoS Biol. 2005, 3, e338. 16167846 10.1371/journal.pbio.0030338 PMC1233413

[30] R. Parsons , R. Parsons , N. Garner , H. Oster , O. Rawashdeh , Bioinformatics 2020, 36, 1208. 31588519 10.1093/bioinformatics/btz730

[31] L. V. M. de Assis , L. Harder , J. T. Lacerda , R. Parsons , M. Kaehler , I. Cascorbi , I. Nagel , O. Rawashdeh , J. Mittag , H. Oster , eLife 2022, 11, e79405. 35894384 10.7554/eLife.79405 PMC9391036

[32] H. Ungefroren , I. Thürling , B. Färber , T. Kowalke , T. Fischer , L. V. M. De Assis , R. Braun , D. Castven , H. Oster , B. Konukiewitz , U. F. Wellner , H. Lehnert , J.‐U. Marquardt , Cancers 2022, 14, 2057. 35565186 10.3390/cancers14092057 PMC9101310

[33] A. J. Cortés‐Espinar , N. Ibarz‐Blanch , J. R. Soliz‐Rueda , B. Bonafos , C. Feillet‐Coudray , F. Casas , F. I. Bravo , E. Calvo , J. Ávila‐Román , M. Mulero , Antioxidants (Basel) 2023, 12, 1606. 37627601 10.3390/antiox12081606 PMC10452039

[34] W. Gadacha , M. Ben‐Attia , D. Bonnefont‐Rousselot , E. Aouani , N. Ghanem‐Boughanmi , Y. Touitou , Redox Rep. 2009, 14, 154. 19695122 10.1179/135100009X466131

[35] V. Arreaza‐Gil , H. Palacios‐Jordan , M. D. M. Romero , C. Torres‐Fuentes , M. A. Rodríguez , X. Remesar , J.‐A. Fernández‐López , A. Arola‐Arnal , Food Funct. 2023, 14, 6941. 37432474 10.1039/d3fo01551c

[36] F. De Chiara , S. Heebøll , G. Marrone , C. Montoliu , S. Hamilton‐Dutoit , A. Ferrandez , F. Andreola , K. Rombouts , H. Grønbæk , V. Felipo , J. Gracia‐Sancho , R. P. Mookerjee , H. Vilstrup , R. Jalan , K. L. Thomsen , J. Hepatol. 2018, 69, 905. 29981428 10.1016/j.jhep.2018.06.023

[37] P. L. Eriksen , H. Vilstrup , K. Rigbolt , M. P. Suppli , M. Sørensen , S. Heebøll , S. S. Veidal , F. K. Knop , K. L. Thomsen , Liver Int. 2019, 39, 2094. 31386258 10.1111/liv.14205

[38] S. Ajaz , M. J. McPhail , L. Gnudi , F. M. Trovato , S. Mujib , S. Napoli , I. Carey , K. Agarwal , Mitochondrion 2021, 57, 119. 33387664 10.1016/j.mito.2020.12.010

[39] A. P. Sousa , D. M. Cunha , C. Franco , C. Teixeira , F. Gojon , P. Baylina , R. Fernandes , Metabolites 2021, 11, 835. 34940595 10.3390/metabo11120835 PMC8703345

[40] S. Sato , K. A. Dyar , J. T. Treebak , S. L. Jepsen , A. M. Ehrlich , S. P. Ashcroft , K. Trost , T. Kunzke , V. M. Prade , L. Small , A. L. Basse , M. Schönke , S. Chen , M. Samad , P. Baldi , R. Barrès , A. Walch , T. Moritz , J. J. Holst , D. Lutter , J. R. Zierath , P. Sassone‐Corsi , Cell Metab. 2022, 34, 329. e8. 35030324 10.1016/j.cmet.2021.12.016 PMC13189211

[41] I. Ginés , K. Gil‐Cardoso , J. Serrano , À. Casanova‐Martí , M.t. Blay , M. Pinent , A. Ardévol , X. Terra , Nutrients 2018, 10, 315. 29518911 10.3390/nu10030315 PMC5872733

[42] Y. Kalender , M. Yel , S. Kalender , Toxicology 2005, 209, 39. 15725512 10.1016/j.tox.2004.12.003

[43] H. Kobayashi , Y. Tanaka , K. Asagiri , T. Asakawa , K. Tanikawa , M. Kage , M. Yagi , Phytomedicine 2010, 17, 197. 20092986 10.1016/j.phymed.2009.12.006

[44] A. Baranwal , P. Aggarwal , A. Rai , N. Kumar , Mini Rev. Med. Chem. 2022, 22, 821. 34477517 10.2174/1389557521666210902162120

[45] Y. Jiang , S. Ding , F. Li , C. Zhang , D. Sun‐Waterhouse , Y. Chen , D. Li , J. Funct. Foods 2019, 62, 103558.

[46] S. Reagan‐Shaw , M. Nihal , N. Ahmad , FASEB J. 2008, 22, 659. 17942826 10.1096/fj.07-9574LSF

[47] Y. Zhang , M. Chen , Y. Zhou , L. Yi , Y. Gao , L. Ran , S. Chen , T. Zhang , X. Zhou , D. Zou , B. Wu , Y. Wu , H. Chang , J. Zhu , Q. Zhang , M. Mi , Mol. Nutr. Food Res. 2015, 59, 1443. 25943029 10.1002/mnfr.201500016

[48] P. Rajan , P. Natraj , S. S. Ranaweera , L. A. Dayarathne , Y. J. Lee , C.‐H. Han , Food Res. Int. 2022, 162, 112059. 36461387 10.1016/j.foodres.2022.112059

