# Supplementary material for: Grape‐Seed Proanthocyanidin Extract (GSPE) Modulates Diurnal Rhythms of Hepatic Metabolic Genes and Metabolites, and Reduces Lipid Deposition in Cafeteria‐Fed Rats in a Time‐of‐Day‐Dependent Manner
Source: Mol Nutr Food Res. 2024 Nov 11;68(23):2400554. doi: 10.1002/mnfr.202400554 (PMC11653167; doi:10.1002/mnfr.202400554)
Supplement: Supplementary file 1 — Supporting Information [file MNFR-68-2400554-s005.pdf]

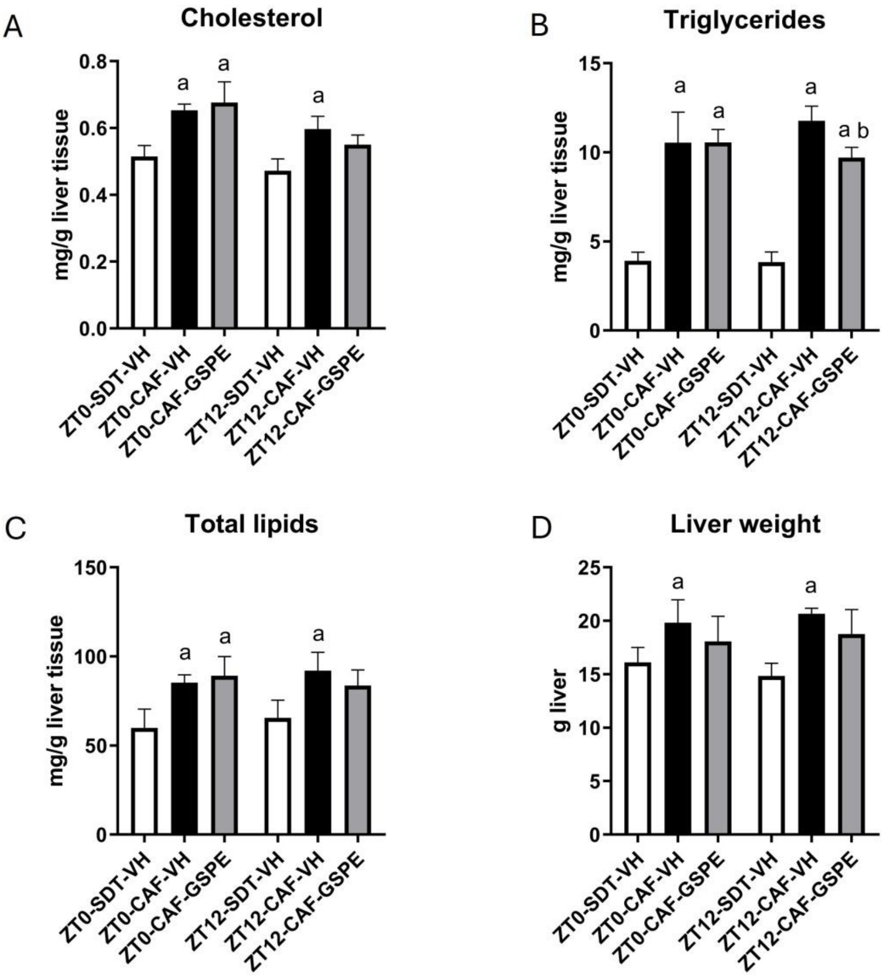

Supplementary figure 1. Liver lipid profiling and weight. A) Hepatic cholesterol in ZT0 and ZT12 experimental groups B) Hepatic triglycerides in ZT0 and ZT12 experimental groups C) Total lipids in ZT0 and ZT12 experimental groups. D) Liver weights in ZT0 and ZT12 experimental groups. Letters indicate significant differences, being “a” difference versus STD-VH groups and “b” difference versus CAF-VH groups. (ANOVA; Tukey post-hoc test; $p<0.05$). Shown are means ± SEM (n=16/group).

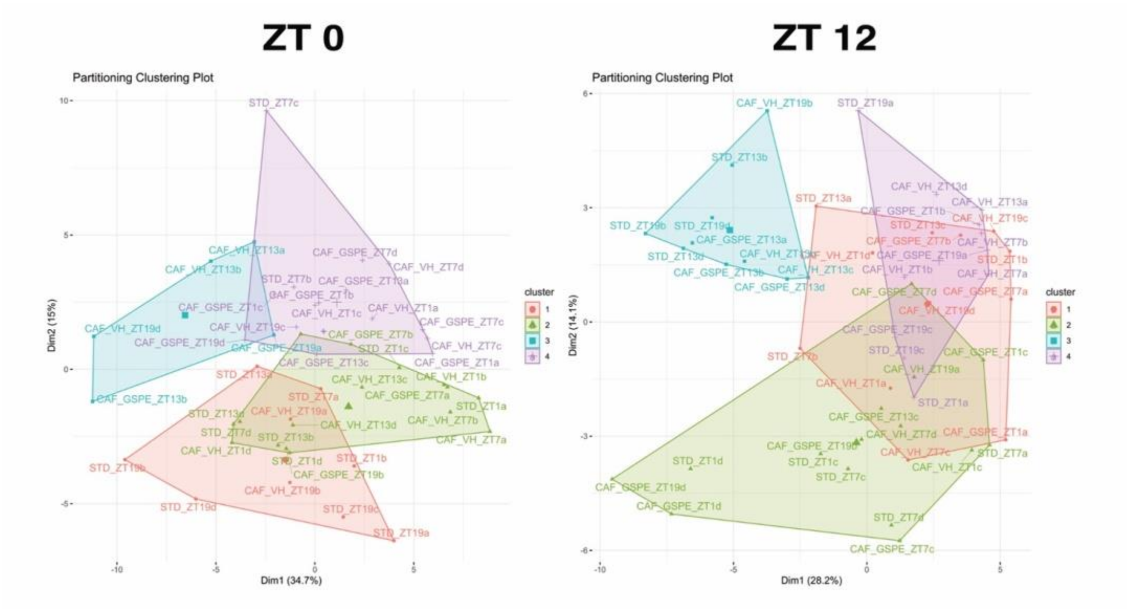

Supplementary Figure 2: Principal component analysis (PCA) of liver metabolome analysis. Rats were fed a STD or CAF diet and received a daily dosage of vehicle or GSPE at the beginning of the light phase (ZT0) (A) or at the beginning of the dark phase (ZT12) (B). Analyzes were performed using the factoextra package and Hartigan-Wong, Lloyd, and Forgy MacQueen algorithms (version 1.0.7) in R.
