# Supplementary material for: Grape‐Seed Proanthocyanidin Extract (GSPE) Modulates Diurnal Rhythms of Hepatic Metabolic Genes and Metabolites, and Reduces Lipid Deposition in Cafeteria‐Fed Rats in a Time‐of‐Day‐Dependent Manner
Source: Mol Nutr Food Res. 2024 Nov 11;68(23):2400554. doi: 10.1002/mnfr.202400554 (PMC11653167; doi:10.1002/mnfr.202400554)
Supplement: Supplementary file 5 — Supporting Information [file MNFR-68-2400554-s002.pdf]

Table S4 - Quantification of rhythm amplitude, phase, and period across conditions in the *in vitro* model with comparative statistical analysis.
